# ChainLineNet: Deep-Learning-Based Segmentation and Parameterization of Chain Lines in Historical Prints

**DOI:** 10.3390/jimaging7070120

**Published:** 2021-07-19

**Authors:** Aline Sindel, Thomas Klinke, Andreas Maier, Vincent Christlein

**Affiliations:** 1 Pattern Recognition Lab, Friedrich-Alexander-Universität Erlangen-Nürnberg (FAU), 91058 Erlangen, Germany; andreas.maier@fau.de (A.M.); vincent.christlein@fau.de (V.C.); 2 Cologne Institute of Conservation Sciences (CICS), Technische Hochschule Köln, 50678 Köln, Germany; thomas.klinke@th-koeln.de

**Keywords:** line segmentation, line detection, line parameterization, generative adversarial networks, Fourier transform, differentiable line fitting, chain lines, paper structure, historical prints

## Abstract

The paper structure of historical prints is sort of a unique fingerprint. Paper with the same origin shows similar chain line distances. As the manual measurement of chain line distances is time consuming, the automatic detection of chain lines is beneficial. We propose an end-to-end trainable deep learning method for segmentation and parameterization of chain lines in transmitted light images of German prints from the 16th Century. We trained a conditional generative adversarial network with a multitask loss for line segmentation and line parameterization. We formulated a fully differentiable pipeline for line coordinates’ estimation that consists of line segmentation, horizontal line alignment, and 2D Fourier filtering of line segments, line region proposals, and differentiable line fitting. We created a dataset of high-resolution transmitted light images of historical prints with manual line coordinate annotations. Our method shows superior qualitative and quantitative chain line detection results with high accuracy and reliability on our historical dataset in comparison to competing methods. Further, we demonstrated that our method achieves a low error of less than 0.7 mm in comparison to manually measured chain line distances.

## 1. Introduction

**Figure 1:**
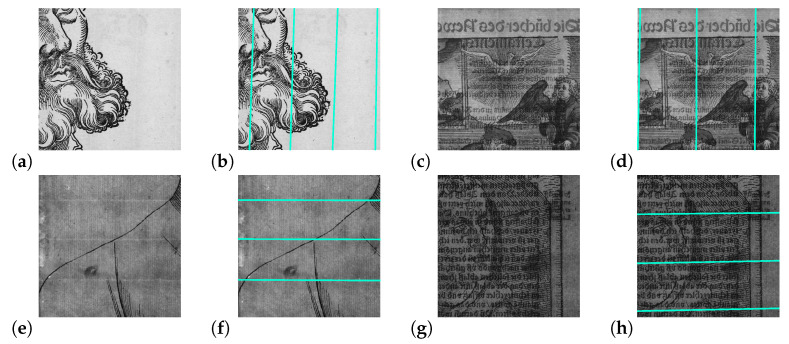
The paper structure in historical prints consists of chain and laid lines, which are perpendicular to each other. Examples using transmitted light photography (**a**,**c**) for vertical and (**e**,**g**) for horizontal chain lines are shown. Our ChainLineNet effectively detects the chain lines (**b**,**d**,**f**,**h**); even so, these are partly occluded by the ink of the artworks. Detail images: (**a**) Hans Sebald Beham, *Martin Luther as Junker Jörg*, Woodcut, Germanisches Nationalmuseum Nürnberg, H1933; (**c**) Unknown, *Martin Luther*, Woodcut, Landesbibliothek Coburg, P I 6/12; (**e**) Lucas Cranach the Elder, *Martin Luther as Junker Jörg*, Woodcut, Klassik StiftungWeimar, Bestand Museen, DK 181/83; (**g**) Hans Baldung Grien, *Martin Luther as Augustinian monk*, Woodcut, Klassik Stiftung Weimar, Herzogin Anna Amalia Bibliothek, Aut. Luther 1520:64; images captured by Thomas Klinke; all rights reserved by the respective museum/library.

Since ancient times, paper has played a prominent role as a carrier for information. In the 16th Century, the only available paper was laid paper, which was manually produced in paper mills. Wood, old rags, and other ingredients were stamped and macerated in water into a pulp of fibers. Then, the paper was scooped by hand using a mold with a wire sieve made of closely spaced “laid” wires and perpendicular more widely spaced “chain” wires. After scooping the fibers from the vat, the remaining fibrous web on the wire sieve forms the paper [1]. On its surface, the grid pattern of the wires is imparted, as can be seen in the transmitted light photographs in Figure 1a,c,e,g. In addition, a watermark can be embedded into the paper structure as a seal of quality and origin by placing bent metal wires on the sieve. Concerning laid paper, the distances between the parallel chain lines vary across the sieve, but are approximately 25–30 mm [2]. For every mold, the chain lines form a unique pattern. Papers created by the same mold show a similar pattern of chain line distances. The impression of the sieve provides a unique conclusion to identify the mold. Images formed by the same mold are called moldmates [1]. Papers from different origins have different line sequences. Characteristics of the paper structure, such as the shape and placement of watermarks, chain line intervals, and the density of laid lines provide possibilities for computer vision to support art historical research. Apart from analyzing the motif itself, e.g., concerning the degree of wear, also, chain line distances can give hints about dating, author assignment, and the chronology of writings and prints [3]. For further refinements, chain line intervals can be analyzed in combination with the density of laid lines, watermarks, and histological findings on the fibers. Traditionally, chain line distances are manually measured by art technologists during the examination and visual inspection of the individual prints, which is very time consuming.

In this paper, we propose an end-to-end trainable method for segmentation and parameterization of chain lines in transmitted light images of German prints from the 16th Century. Our method exploits the power of deep neural networks in combination with prior knowledge from image and signal processing. We trained a conditional generative adversarial network by using a multitask loss for line segmentation and line parameterization. For the estimation of line coordinates, we designed a fully differentiable pipeline that comprises the steps of line segmentation, horizontal alignment and 2D Fourier filtering of line segments, line region proposals, and differentiable line fitting. For training and evaluation, we created a dataset of high-resolution transmitted light images of historical prints for which we manually annotated line coordinates. Our ChainLineNet learns to detect the chain lines with high reliability even if there are interferences caused by watermarks or if the lines are partly occluded by the ink of the artwork; cf. Figure 1.

## 2. Related Work

To digitize the paper structure of historical prints, several imaging techniques, e.g., beta-radiography, transmitted light photography, transmitted infrared, or thermography, can be applied. Transmitted light photography is a very fast application, inexpensive, and very easy to handle. Hence, additional image processing might be necessary due to interferences such as ink that remain visible. These interferences disappear in the images using the other modalities, but especially beta-radiography is only applicable for large institutions due to the necessary technical and financial input.

### 2.1. Segmentation and Detection of Chain Lines

There are a few approaches for the automated segmentation of chain lines of paper. Van der Lubbe et al. [3] assumed straight and vertical chain lines for chain line detection in radiography. They used uniform filtering and morphological opening and closing operators as the preprocessing and applied a vertical projection to detect the vertical lines as peaks of the projection. Atanasiu [4] proposed a software measurement tool to analyze the density of laid lines by using the bidimensional discrete fast Fourier transform. In a preprocessing step, an emboss edge-enhancing high-pass filter reduces noise; however, the orientation of the laid lines has to be determined beforehand. Van Staalduinen et al. [5] presented an approach for moldmate matching using the specific paper features of chain and laid lines. The lines are detected by means of the shadow around the chain lines. The sequences of line distances for moldmate matching are computed with a combination of the discrete Fourier transform and Radon transform based on the assumption of straight and equidistant lines. Hiary et al. [2] focused on the digitization, extraction, and graphical representation of watermarks. They used backlight scanning and image processing such as mathematical morphological operations to automatically extract and convert watermarks to graphical representations. In an intermediate step, they rotated the image to upright the chain lines by means of chain line detection and Radon transform. Johnson et al. [1] published a method to find moldmates among Rembrandt’s prints in beta-radiographs. Their chain line pattern matching approach uses unique chain spacing sequences in the paper structure rather than watermarks to identify the moldmates. Based on the assumption of straight, but not necessarily parallel lines, they rotated the chain lines to the vertical and obtained the angle of rotation by applying the Radon transform. Finally, the lines were detected using a vertical filter and the Hough transform.

In our previous work [6], we trained a convolutional neural network (CNN) to automatically segment the chain lines in artworks. Therefore, we employed the UNet [7] as the network architecture and proposed two postprocessing steps by employing either random sample consensus (RANSAC) [8] or the Hough transform to locate and parameterize complete lines in the binarized segmentation results. First, we determined the global orientation of the lines (horizontal or vertical) based on applying the Sobel filter. For the RANSAC-based approach, we extracted line segments from the segmentation mask using connected components and filtered out too small or falsely oriented line segments. The remaining line segments were grouped using agglomerative clustering, and RANSAC was utilized to fit lines through each group of points. For the Hough-based approach, we applied Hough voting on the segmentation masks and used agglomerative clustering to merge line predictions.

### 2.2. Segmentation and Detection of Lines

Looking more generally at the task of line detection in the fields of wireframe detection and semantic and horizon line detection, deep learning has been extensively applied.

Wireframe detection is the detection of line segments and junctions in a scene to describe all kinds of geometric objects or architectures [9]. Huang et al. [9] proposed a two-stage method that predicts heat maps for the line segments and junctions using two CNNs and combines junctions and lines by applying several postprocessing steps. To train their method, they created a large wireframe benchmark dataset. Zhou et al. [10] designed an end-to-end trainable L-CNN that directly predicts vectorized wireframes. The L-CNN consists of a stacked hourglass network as the feature extraction backbone, a heat-map-based junction proposal module, a line-sampling module that generates line candidates based on the predicted junctions, and a line verification module, for which the line of interest (LoI) pooling layer is utilized, which compares line segments with corresponding positions in the feature maps of the backbone. The holistically-attracted wireframe parser (HAWP) [11] was built on the L-CNN and introduced a novel line segment reparameterization by using a holistic attraction field map that assigns each pixel to its closest line segment. Lin et al. [12] proposed in their deep Hough transform line priors method to combine line priors with deep learning by incorporating a trainable Hough transform block into a deep network and performing filtering in the Hough domain with local convolutions. For the application of line detection on the Wireframe datasets, they used the L-CNN [10] and the HAWP [11] as backbones and replaced the hourglass blocks with their Hough transform blocks.

For the application of semantic lines or horizon detection in natural scenes, Lee et al. [13] proposed the VGG16-based semantic line network (SLNet) with line pooling layers, which combines line detection as a multitask loss of classification and regression. The deep Hough transform method by Zhao et al. [14] incorporates the Hough transform into a one-shot end-to-end learning pipeline by using a CNN encoder with feature pyramids for feature extraction and performing the line detection in Hough space. Nguyen et al. [15] transferred the ideas from object detection to design the LS-Net for power line detection that uses a CNN with two heads: one for classification and the other for line regression. Brachmann et al. [16] combined neural guidance with differentiable RANSAC (DSAC) [17] for horizon line estimation.

### 2.3. Contour Detection Using Generative Adversarial Networks

Another related group to our chain line segmentation method consists of contour detection methods using generative adversarial networks (GANs), as the chain lines and contours have a similar shape, and hence, both are sparse segmentation tasks. Contour detection datasets usually contain multiple ground truth annotations per image by different annotators, since the amount of annotated lines differs between the annotators depending on the subjective decision of the individual annotator whether a contour is important enough to be drawn. ContourGAN [18] uses a conditional GAN with a VGG16-based generator network for contour detection in natural images. The adversarial loss is combined with a binary cross-entropy content loss for which the set of ground truth contour images is linearly merged into a single ground truth image. Art2Contour [19] utilizes a conditional GAN with a ResNet-based generator network for salient contour detection in prints and paintings. Art2Contour is trained with a combined loss of the cGAN loss and a task loss consisting of multiple regression terms, which separately treat the single ground truth images. Our method was based on the network architecture used by Art2Contour, but we introduced a novel multitask loss to simultaneously learn line segmentation and line parameterization.

## 3. Method

**Figure 2:**
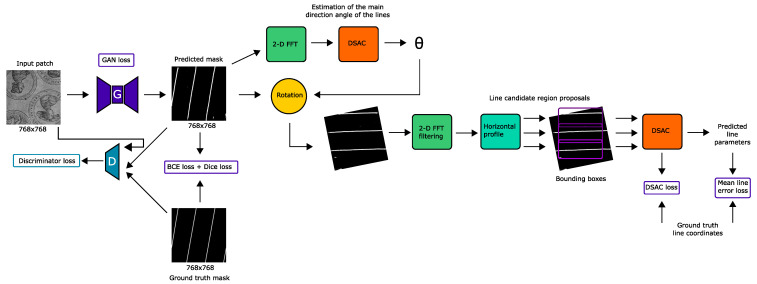
ChainLineNet: End-to-end trainable segmentation and parameterization of chain lines using a conditional generative adversarial network-based approach. The generator network is trained using a multitask loss consisting of the segmentation task and the line parameterization task. We propose a fully differentiable pipeline for line coordinates’ estimation that is composed of line segmentation, primary line orientation prediction, horizontal alignment of the lines, 2D Fourier filtering, line region proposals, and line fitting with differentiable sample consensus (DSAC) [17]. Detail transmitted light image (input patch): Unknown, *Compilation sheet with round portraits*, Woodcut, Kupferstichkabinett, Staatliche Museen zu Berlin, 44-1884; captured by Thomas Klinke; all rights reserved by the respective museum.

Our proposed method for the segmentation and detection of chain lines in transmitted light images of historical prints is illustrated in Figure 2. In this section, we introduce the network architecture, the end-to-end trainable pipeline, the loss functions, and inference.

### 3.1. Chain Line Segmentation Network Architecture

Our chain line segmentation network is a conditional generative adversarial network (cGAN) [20] consisting of a generator and discriminator network. Our generator network is the ResNet-based [21] encoder–decoder architecture that was introduced for style transfer [22], having ResNet blocks in the bottleneck, and in contrast to UNet [7], it does not have skip connections between the encoder and decoder [19,23]. As the discriminator network, we used a regular global GAN that has been shown to be effective for contour detection [19,23].

### 3.2. End-to-End Training of Line Segmentation and Parameterization

We jointly trained the generator network for the tasks of line segmentation and line parameterization in an end-to-end fashion by only using differentiable modules and functions inspired by known operator learning [24], while the discriminator network only evaluates the segmentation output against the ground truth segmentation mask.

In generative adversarial networks (GANs), the generator network and the discriminator network are alternately optimized. The generator *G* is fed with a random noise vector *z* to generate the output image *y*, while the discriminator *D* is trained to distinguish real images from fake images. In the case of conditional GANs, the output of the generator *y* is additionally conditioned to an input, e.g., an image *x*. Thus, the generator is trained to generate realistic-looking images that are directly related to the input images. The objective function of cGAN is formulated as:

(1) $$\begin{array}{c} \begin{array}{cc} L_{\mathrm{cGAN}}\left(x,y,z\right) & =\min_{G}\max_{D}E_{x,y}\left[\log D(x,y)\right]\\ \; & +E_{x,z}\left[\log\left(1-D(x,G(x,z)\right)\right]\;. \end{array} \end{array}$$

The cGAN principle can be directly applied to the line segmentation task. The generator *G* learns to produce precise line segmentation masks $y\in R^{s_{1}\times s_{2}}$ for the input artwork images $x\in R^{s_{1}\times s_{2}}$, encouraged by the discriminator *D*, which learns to detect those fake ones. The cGAN loss is generally combined with a task loss. We extended this approach by also including the line coordinates’ estimation process for the generator task loss:

(2) $$\begin{array}{cc} L_{G}\left(x,y,g,h_{1},\cdots ,h_{m},p,q\right) & =L_{\mathrm{cGAN}}\left(x,y\right)+\lambda_{0}\;L_{\mathrm{Task}}\left(y,g,h_{1},\cdots ,h_{m},p,q\right)\;, \end{array}$$

where $g\in R^{s_{1}\times s_{2}}$ is the ground truth segmentation mask, $\left\{h_{1},\cdots ,h_{m}\right\}$ the line hypotheses sampled for DSAC, $p\in R^{M\times 4}$ the predicted line coordinates, and $q\in R^{N\times 4}$ the ground truth lines coordinates with $\left\{x_{0}^{i},y_{0}^{i},x_{1}^{i},y_{1}^{i}\right\}$ being the start and end points of the lines. Our multitask loss is defined as the weighted sum of the line segmentation task and line parameterization task:

(3) $$\begin{array}{cc} L_{\mathrm{Task}}\left(y,g,h_{1},\cdots ,h_{m},p,q\right) & =\lambda_{\mathrm{BCE}}L_{\mathrm{BCE}}\left(y,g\right)+\lambda_{\mathrm{DICE}}L_{\mathrm{DICE}}\left(y,g\right)\\ \; & +\lambda_{\mathrm{DSAC}}L_{\mathrm{DSAC}}\left(h_{1},\cdots ,h_{m},q\right)+\lambda_{\mathrm{MLE}}L_{\mathrm{MLE}}\left(p,q\right), \end{array}$$

where $\lambda_{\mathrm{BCE}},\lambda_{\mathrm{DICE}}$ are the weights for the binary cross-entropy loss (BCE) and Dice loss (DICE) for the segmentation task and $\lambda_{\mathrm{DSAC}},\lambda_{\mathrm{MLE}}$ are the weights for the DSAC loss [17] and the mean line distance error loss (MLE) for the line parameterization task.

### 3.3. Line Parameterization Pipeline and Line Loss Functions

The prediction of the line parameters is subdivided into the parts of line segmentation, prediction of the main line orientation to horizontally align the lines, 2D Fourier filtering, line region proposals, and line fitting with differentiable sample consensus (DSAC) [17], as illustrated in Figure 2. As chain lines are nearly parallel to each other and have similar distances between them, we used the 2D fast Fourier transform (FFT) to find the main orientation of the lines in the images. The 2D Fourier representation of the segmentation mask shows the response to the dominant direction of the lines.

**Figure 3:**
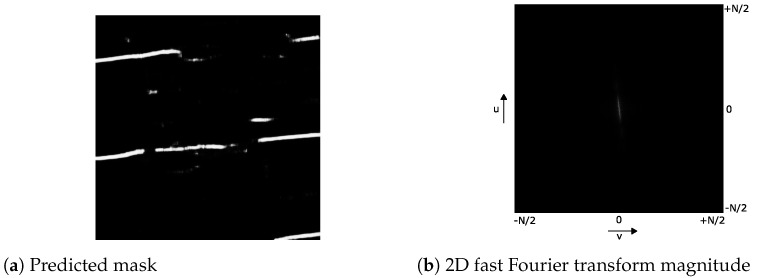
The 2D fast Fourier transform of the predicted segmentation mask in (**b**) shows a centered line whose orientation is orthogonal to the dominant orientation of the line segments in (**a**) the predicted segmentation mask.

As can be seen in the centered 2D Fourier magnitude image in Figure 3, there is one line in the center with an orientation orthogonal to that of the chain lines in the image domain. Hence, we extracted the k=500 points with maximal intensity in the centered magnitude image and fit a line through them using DSAC [17]. Then, we computed the polar angle of the line $\theta_{ft}$ and determined the rotation angle $\theta_{rot}$ to align the lines horizontally by:

(4) $$\theta_{rot}=\left\{\begin{array}{cc} 90^{\circ }-∥\theta_{ft}∥ & \theta_{ft}<0,\\ 90^{\circ }+∥\theta_{ft}∥ & \mathrm{otherwise} \end{array}\right.$$

**Figure 4:**
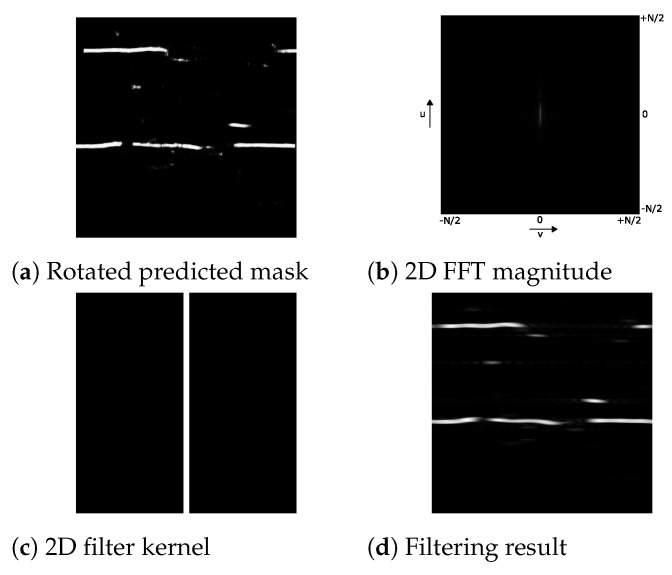
Two-dimensional filtering in Fourier domain to reduce nonhorizontal lines. In (**a**), the horizontally aligned predicted segmentation mask, in (**b**), its 2D FFT magnitude, in (**c**), the 2D filter kernel, and in (**d**), the filtering result of the rotated predicted segmentation mask is shown.

Next, we rotated the predicted segmentation masks, the ground truth segmentation masks, and the ground truth line coordinates; see Figure 4. The segmentation mask can show some line segments of different orientations, for instance due to watermarks, as these have the same intensity as chain lines in the transmitted light images. To reduce nonhorizontal line segments, we applied a vertical filter H(u,v) in the Fourier domain to the rotated segmentation masks F(u,v) with u,v∈{−N/2,N/2}:

(5) $$G\left(u,v\right)=F\left(u,v\right)H\left(u,v\right),H\left(u,v\right)=\left\{\begin{array}{cc} 1 & ∥v∥<\tau ,\\ 0 & \mathrm{otherwise} \end{array}\right.$$

As convolution with a filter kernel in the time domain is elementwise matrix multiplication in the Fourier domain, we can simply multiply the 2D Fourier image with a matrix that has only zero elements except for a vertical band of width 2τ with τ=10 pixels at the center.

**Figure 5:**
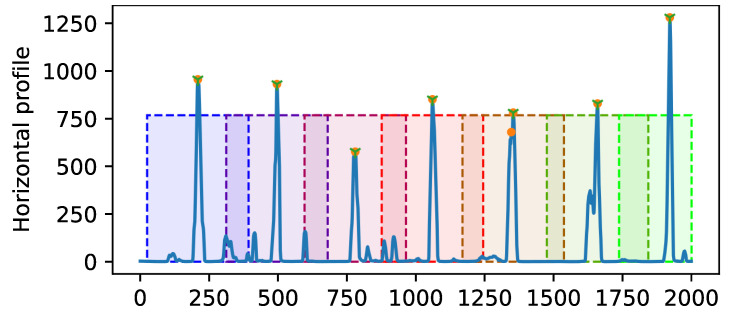
Selection of peaks in the horizontal profile for one example image (length of 2000 pixels) with 7 chain lines from the validation set. The peaks are marked with an orange dot, and the final selection of peaks after distance thresholding are additionally marked with a green cross. Then, a bounding box is placed at the center of each selected peak.

To determine the number of lines and their rough positions, we computed the horizontal profile by summing up all intensity values of the filtered segmentation mask along the *x*-direction (see Figure 5). All segmented lines correspond to peaks in the profile. We applied 1D max-pooling to the profile to obtain all local maxima. To filter out all local maxima that most likely do not belong to the horizontal lines, we applied intensity and spatial distance thresholding. As prior knowledge, we considered that chain lines have approximately the same distances; hence, we first selected the peaks that have a distance of at least 0.75 of the maximal distance of all peaks and then refined the selection by keeping only those that have at least 0.6 of the maximal distance of the selected peaks.

A horizontally oriented bounding box $\left[0,W,y_{i}-D_{\mathrm{thresh}},y_{i}+D_{\mathrm{thresh}}\right]$ is defined for each of the refined peak positions $y_{i}$ using the previously computed threshold $D_{\mathrm{thresh}}$ as the length to both sides. In the case that no peak position can be found or can be selected, we defined one bounding box for the entire image.

Next, we extracted for each bounding box the maximal $k_{p}$ points within the bounding box region of the segmentation mask to use them for line fitting with DSAC. DSAC [17] formulates the hard hypothesis selection of RANSAC as a probabilistic process that allows end-to-end learning. The application of DSAC for line fitting (implementation by Brachmann et al.: https://github.com/vislearn/DSACLine (accessed on 14 July 2021)) consists of the following steps:

1. Line hypothesis sampling: Based on the predicted point coordinates *z*, *m* line hypotheses $\left\{h_{1},\cdots ,h_{m}\right\}$ are randomly sampled by choosing for each hypothesis two points of the point set. Each hypothesis predicts an estimate for the line parameters, the slope *a* and intercept *b* of the line equation y=ax+b;
2. Hypothesis selection: A scoring function s(h) computes a score for each hypothesis based on the soft inlier count. The hypothesis $h_{j}$ is selected according to the softmax probabilistic distribution $P\left(j;z\right)=\frac{\exp(s\left(h_{j}\right))}{\sum_{k}\exp\left(s\left(h_{k}\right)\right)}$;
3. Hypothesis refinement: The hypothesis is refined by using the weighted Deming regression for line fitting [25], which is a special case of the total least-squares that accounts for errors in the observations in both the *x*- and *y*-direction, for which we used the soft inlier scores as the weights.

The DSAC loss function, which we incorporated into our task loss function, is defined as:

(6) $$L_{\mathrm{DSAC}}\left(h_{1},\cdots ,h_{m},q\right)=\sum_{k}\left(\frac{\exp(s\left(h_{j}\right))}{\sum_{k}\exp\left(s\left(h_{k}\right)\right)}∥|p\left(h_{k}\right)-q{∥|}_{2}\right),$$

where $p(h_{k})$ refers to the predicted start and end points for the line hypothesis $h_{k}$ and q refers to the ground truth start and end points. The start and end points of the lines are determined as the intersection with the image borders.

Since we applied DSAC to each bounding box region separately and the bounding box positions are determined automatically based on the segmentation output of the network, we needed to assign one ground truth line to each bounding box. We distinguish three cases: (1) If there is only one ground truth line inside the bounding box region, this one is selected. (2) If the region contains multiple ground truth lines, we chose the longest line. (3) Lastly, if there is no ground truth line inside the region, we selected the line with the minimal distance of its start and end points to the borders of the region.

The DSAC loss minimizes the distance of the predicted lines to the closest ground truth lines; however, if too few bounding boxes are predicted, some ground truth lines will not be included. To account for these false negatives, we defined a second line loss term, the MLE loss, that picks for each ground truth line the closest predicted line of the best hypothesis $h_{j}$ and computes the mean error:

(7) $$L_{\mathrm{MLE}}\left(p,q\right)=\frac{1}{N}\sum_{i}\min\left(D_{i}\right),\;D=cdist\left(p,q\right),$$

where $D\in R^{N\times M}$ is the Euclidean distance between each pair of the two collections of row vectors of $p\in R^{M\times 4}$, $q\in R^{N\times 4}$, and $D_{i}$ is the $i^{th}$ row of the distance matrix.

### 3.4. Inference of Chain Line Segmentation and Parameterization Network

Since the network architecture is fully convolutional, the complete images are fed to the GAN and are processed in the same manner as for training, resulting in the line predictions of the rotated image. Hence, to obtain the final line coordinate predictions of the original image, the inverse rotation is applied to the predicted lines.

## 4. Experiments and Results

In this section, we describe our dataset for chain line detection in historical prints, we evaluate the performance of our method for line segmentation and line parameterization, and compare it to the state-of-the-art methods and to manual line measurements.

### 4.1. Chain Line Dataset

The dataset consists of high-resolution grayscale transmitted light images of prints from the 16th Century, including portraits of Martin Luther and contemporaries. For our dataset, we selected in total 95 images in which the chain lines were recognizable by the human eye. All images contain chain lines that are either horizontally or vertically distributed at approximately the same distances.

**Figure 6:**
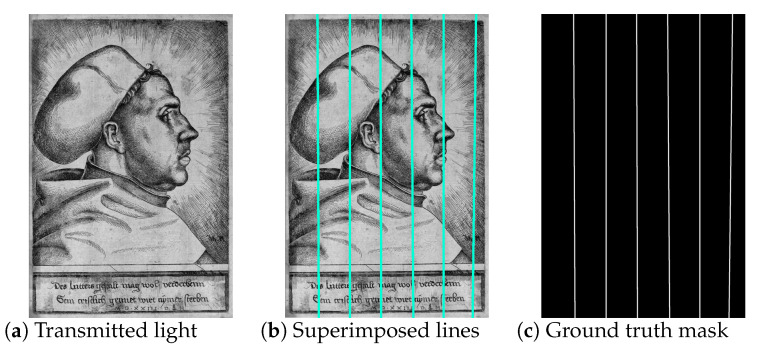
Illustration of the line annotation (**a**) in the transmitted light images of historical prints by (**b**) selecting start and end points of the lines and (**c**) computing the corresponding mask images. Image: (**a**) Daniel Hopfer, *Martin Luther with the doctor’s cap*, Etching, Germanisches Nationalmuseum Nürnberg, K722; captured by Thomas Klinke; all rights reserved by the respective museum.

We manually annotated the chain lines in the images by selecting two points on each line and fitted a straight line through them, as illustrated in Figure 6a,b. We used the *x* and *y* coordinates of the start and end points, as well as the corresponding mask images (Figure 6c) that contain the segmented ground truth lines as labels for training, validation, and testing.

The sharp edges of the annotated lines in the mask images are smoothed by applying a Gaussian filter with a standard deviation of 3. The images are divided into 35 images for training, 12 images for validation, and 48 images for testing. The images were acquired at a very high resolution with image sizes up to 5000×6500 pixels. Since chain lines are very fine structures that are difficult to detect, the highest possible image resolution is recommended, but is limited by hardware constraints. To be able to feed the entire image at once for inference using one Nvidia Titan XP GPU (NVIDIA Corporation, Santa Clara, CA, USA), we scaled all images to the maximal length of 2000 pixels, which is sufficient for the chain line detection task. To train the neural network, we split the scaled images of the training and validation set into image patches of size 768×768 pixels with an overlap stride of 384. The image patches contain between one and five lines per patch. Patches that do not contain any line were excluded from training. Further, we applied offline data augmentation (see below) to double the number of training and validation patches, resulting in 1150 training and 370 validation patches.

### 4.2. Implementation Details

Our method was implemented using the PyTorch framework, and the end-to-end training and inference both ran completely on the GPU. The generator network (9 ResNet blocks) and the discriminator network were trained from scratch for 100 epochs with early stopping by using the Adam optimizer, a learning rate of η=0.0002 with linear decay to 0 starting at Epoch 50, momentum (0.5,0.999), a batch size of 2, $\lambda_{0}=1000$ [19], $\lambda_{BCE}=0.5$, $\lambda_{DICE}=0.5$, $\lambda_{DSAC}=0.5$, and $\lambda_{MLE}=0.5$. For DSAC, m=64 hypotheses are sampled based on $k_{p}=500$ points from each bounding box per patch or $k_{p}=1300$ points from each bounding box per image.

Prior to training, we augmented our training and validation set in an offline manner with rotated images, i.e., rotations by 90 degrees were applied to produce the same number of vertical and horizontal lines. During training, we applied online data augmentation (color jittering, blurring, horizontal and vertical flipping, and rotation with angles uniformly sampled in the range of $\left(-20,20\right)$ degrees) only to the training set, and not to the validation set.

### 4.3. Evaluation of Line Segmentation

In this section, we compare different architectures for the task of chain line segmentation using pixelwise precision, recall, and the Dice coefficient (i.e., pixelwise F1-score) of the predicted segmentation results and ground truth segmentations. To compute the metrics, we applied a threshold of 0.5 to binarize the segmentation masks. For this experiment, all networks were trained only for the segmentation task (i.e., $\lambda_{BCE}=\lambda_{DICE}=0.5$, $\lambda_{DSAC}=\lambda_{MLE}=0$). We compared the UNet (with feature dimension F=16; 1,942,289 parameters, and F=64; 31,036,481 parameters) and the ResNet-based encoder–decoder architecture (F=64; 11,370,881 parameters) alone and plugged into the generative adversarial training as generator networks. As summarized in Table 1 for the validation set, all network architectures achieve higher recall than precision. Precision is highest for the small UNet-GAN and recall for the ResNet-GAN, directly followed by the ResNet encoder–decoder (ResNet-E-D). The Dice coefficient, which combines the pixelwise precision and recall into one measure, is also highest for the ResNet-GAN and second best for the ResNet encoder–decoder. Concerning the Dice coefficient, UNet seems not to profit from adversarial training in our specific case. Based on these observations, we chose the ResNet-GAN architecture for our end-to-end trainable line segmentation and detection method.

### 4.4. Evaluation of Line Detection and Parameterization

For the evaluation of line detection and parameterization, we compared the number of predicted lines using precision, recall, and the $F_{1}$ score. Therefore, we counted the number of true positives, false positives, and false negatives based on a pixel distance threshold of 50 by computing the distance between the start and end point of the predicted lines and ground truth lines that were manually annotated on the digital images. As a metric, we computed the mean pixel differences of chain line positions w. r. t. the ground truth line coordinates only for the true positive lines. Furthermore, we compared the automatically computed chain line distance intervals with the manual measurement of an art technologist, who has measured the chain line distance intervals directly on the physical paper during his art technological examination. To convert the predicted pixel distance intervals into distance intervals in millimeters such that these can be directly compared to the physical measurements, we scaled the images based on the manually measured width of the artwork. For the chain line distance comparison, we only considered images in which both the number of true positive lines and the total number of detected lines differs only at most by about 2 lines from the number of reference lines by the art technologist. We used cross-correlation to automatically find the best position to arrange the two distance intervals as they can be shifted against each other if one or two lines are not detected. Then, we computed the mean absolute difference of the overlap of both intervals.

#### 4.4.1. Ablation Study

We evaluated the influence of our ChainLineNet using all task loss terms in contrast to setting individual terms to zero. First, we compare the line detection results in Table 2 for the test set. By using our novel multitask loss consisting of the segmentation losses (BCE+DICE) and the line parameterization losses (DSAC+MLE), we achieved a gain in the $F_{1}$ score of about 1% in comparison to training the network only for the segmentation task (ChainLineNet-2) and of about 2% in comparison to the end-to-end training only by using the BCE+DICE+DSAC losses (ChainLineNet-1). The DSAC loss alone does not consider false negatives, hence resulting in a lower recall.

**Figure 7:**
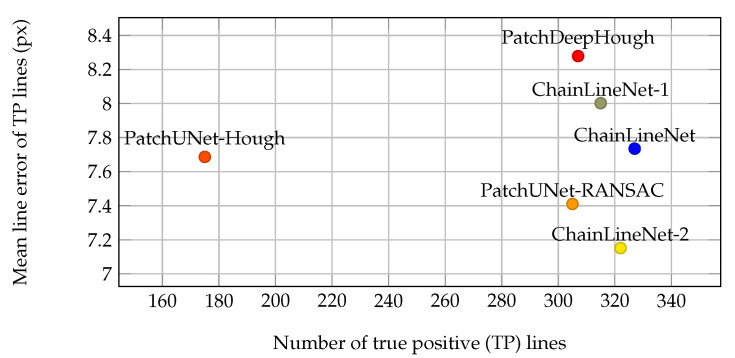
Comparison of the mean pixel line error between the true positive predicted line coordinates and the ground truth line coordinates for the test set. Our ChainLineNet (complete task loss) is compared to the end-to-end training with the task losses BCE+DICE+DSAC (ChainLineNet-1), to the training using only the segmentation task losses BCE+DICE (ChainLineNet-2), and to the competing methods.

Secondly, we compared the difference of the line positions between the predicted and ground truth lines in Figure 7 for the test set. The line error was only calculated for true positives. The mean line error of true positives lies between 7 and 8 pixels with the lowest error for ChainLineNet-2 (only segmentation), followed by ChainLineNet (all losses) and ChainLineNet-1 (segmentation + DSAC). However, the results are very close, and the number of true positives of the ChainLineNet is a bit higher, which could be a reason for the slightly higher pixel error of almost 0.6 in comparison to ChainLineNet-2.

**Figure 8:**
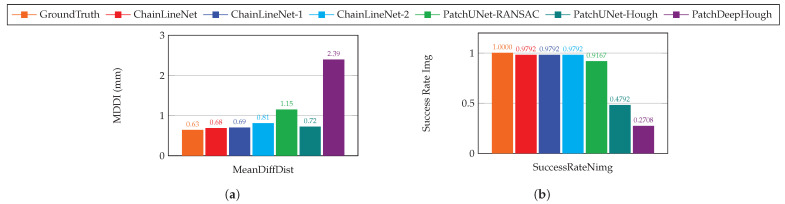
Comparison of (**a**) the mean difference of distance intervals (MDDI) between the predicted distances and the reference distances (manually measured by an art technologist) and (**b**) the success rate of images for which the distance intervals were compared. Our ChainLineNet (complete task loss) is compared to the end-to-end training with the task losses BCE+DICE+DSAC (ChainLineNet-1), to the training using only the segmentation task losses BCE+DICE (ChainLineNet-2), to the competing methods, and to the ground truth on the test set.

Lastly, we compare in Figure 8, for the test set, the distance intervals for the images that contain a suitable number of lines with the reference distance measurements. For this comparison (see Figure 8b), only one image was excluded, giving a success rate of about 98% for all versions of ChainLineNet. The mean difference of the distance intervals (Figure 8a) is below 1 mm for all three variants, whereas ChainLineNet (all losses) achieves the best result, directly followed by ChainLineNet-1 (with DSAC) and ChainLineNet-2 (only segmentation) being a bit inferior.

#### 4.4.2. Comparison to the State-of-the-Art

In this section, we measure the performance of our ChainLineNet compared to competing methods. We retrained the UNet architecture (F = 16) of our previous work [6], which was implemented in TensorFlow, for our renewed historical print dataset for 30 epochs using a learning rate of η=0.0001 and a batch size of 5. During inference, the UNet was executed patchwise, and two postprocessing methods were applied to the reassembled segmentation output [6], which we refer to as PatchUNet-RANSAC and PatchUNet-Hough. Secondly, we trained the deep Hough transform line prior method [12] for our line detection task, which we abbreviate as PatchDeepHough. The method was originally developed for wireframe detection; thus, some modifications were necessary to make it applicable to our task. We used their offline data augmentation, which quadrupled the number of training patches, and trained the network from scratch for 50 epochs with early stopping using a learning rate of η=0.0004 and a batch size of 4. Due to the high complexity of the voting matrix needed for the Hough transform, we were not able to increase the input size of the network for inference, such that we used the default setting of 512×512 and applied the method patchwise. We added the following postprocessing steps to filter, merge, and extend line segments to full lines: First, we computed the dominant orientation of the line segments, i.e., horizontal or vertical. Then, we excluded all line segments with the opposite orientation and whose Hough score was below 0.7. For the remaining line segments, we walked along the perpendicular direction of the line segments and grouped the segments within a neighborhood of 20 pixels. For each group, we used linear least-squares regression to fit a line through the start and end points of the line segments. In the case of a vertical main orientation of the lines, we switched the *x* and *y* coordinates for line fitting to obtain more accurate results.

The quantitative results for line detection and parameterization for the test set consisting of 48 images and in total 342 correct lines are summarized in Table 2 for precision, recall, and the $F_{1}$-score. ChainLineNet outperformed all machine learning methods with an $F_{1}$-score of 96.9%, precision of 98.2%, and recall of 95.6%, being close to manual measurements, which obtain an $F_{1}$-score of 99.6%. In comparison to PatchUNet-RANSAC, which also performs quite well, we achieved an absolute gain of about 4 in the $F_{1}$-score. PatchDeepHough detects too many false positive lines; thus, it only achieved poor precision and a clearly lower $F_{1}$-score of 70.6%. PatchUNet-Hough detects distinctively less correct lines, resulting in a low recall and the lowest $F_{1}$-score of 62%.

The comparison for the pixel mean line error of true positive lines, depicted in Figure 7, shows that all methods predict the line coordinates comparably accurately with an error between 7.2 and 8.3 pixels. The result of ChainLineNet with 327 out of 342 correct lines is the most reliable, as most lines were used to compute the mean line error.

Next, we compare the chain line distance intervals to the reference measurements in Figure 8. The chain line distance intervals computed using ChainLineNet for 47 out of 78 test images only differ by 0.68 mm from the reference intervals, which is an excellent result, when we consider that the comparison of the manually annotated ground truth lines and the reference lines differs by 0.63 mm. Plausible reasons for the measurement inaccuracies are the conversion of the images of the artworks to millimeters, the fact that the location where the line distances are measured can differ between manual and digital measurements, and that chain lines are approximated as straight lines. The other tested machine learning methods show less precision for the distance interval computation. PatchUNet-Hough has a slightly higher mean difference, but only less than half of the images are suitable for the comparison (see Figure 8b). PatchUNet-RANSAC has a slightly lower success rate than ChainLineNet with their mean difference lying just above 1 mm. PatchDeepHough performs worst. With only a success rate of 27% of the images, their mean difference is above 2 mm.

**Figure 9:**
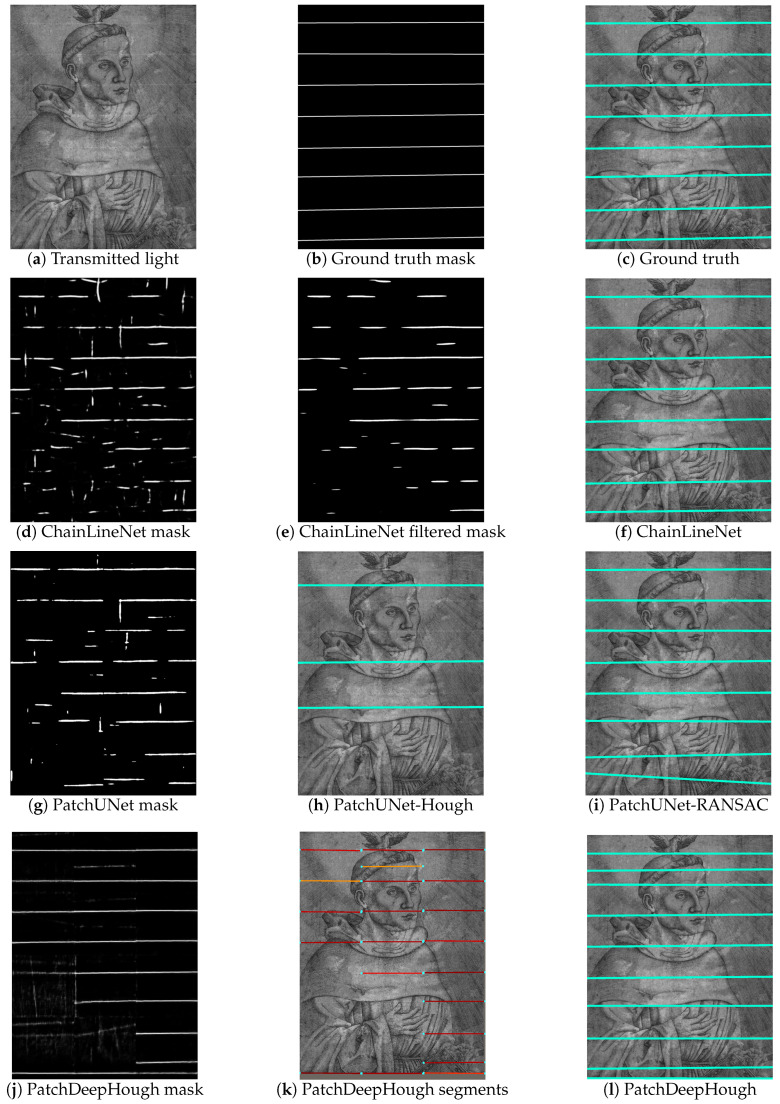
Qualitative results of the chain line detection for one historical print containing horizontal chain lines. Transmitted light image: Hieronymus Hopfer, *Martin Luther as Augustinian monk with Holy Spirit as a dove*, Etching, British Museum, London, 1845-0809-1486; Photo © Thomas Klinke, courtesy of the Trustees of the British Museum; all rights reserved by the respective museum.

**Figure 10:**
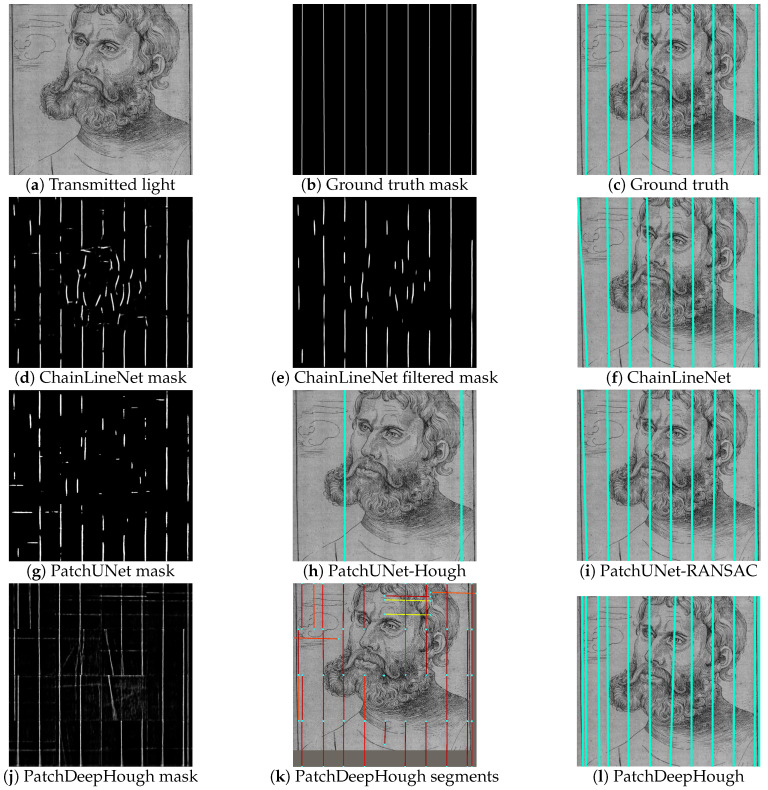
Qualitative results of the chain line detection for one historical print containing vertical chain lines and a watermark. Transmitted light image (detail): After Lucas Cranach the Elder, *Martin Luther as Junker Jörg*, Collotype, Kunstsammlungen der Veste Coburg, H.0064; captured by Thomas Klinke; all rights reserved by the respective museum.

The qualitative results are shown in Figure 9 for one example with horizontal chain lines and in Figure 10 for an example with vertical chain lines. For both figures, the transmitted light image of the artwork, the ground truth segmentation mask, and the ground truth lines superimposed on the artwork are depicted in the first row. Figure 9d and Figure 10d show the raw segmentation outputs of the ChainLineNet that contain line segments and noise. The noise is reduced in Figure 9e and Figure 10e by 2D Fourier filtering. Here, the filtered mask images are binarized for visualization, because only the points with maximal intensity are selected for DSAC. In Figure 9f and Figure 10f, the final line parameterization results of ChainLineNet are shown, which are in high accordance with the ground truth lines. Figure 9g and Figure 10g show the binarized segmentation output of PatchUNet that is also composed of line segments and noise. Two different postprocessing approaches are applied to the PatchUNet output. PatchUNet-Hough (Figure 9h and Figure 10h) detects clearly fewer lines than PatchUNet-RANSAC (Figure 9i and Figure 10i). The grayscale heat map of PatchDeepHough in Figure 9j and Figure 10j shows many clear lines, but also areas of uncertainty. Due to the patchwise application, line segments are separately fitted in each patch (Figure 9k and Figure 10k), where the Hough voting score is indicated by the line segment color ranging from low (blue) to high (red). PatchDeepHough predicts clearly too many lines, as can be seen in Figure 9l and Figure 10l. Despite the watermark that is included in the paper structure of Figure 10a, all methods are able to detect chain lines that interfere with the watermark.

Overall, our method achieves excellent performance, but there are some limitations. In the case of bent wires, our method cannot determine the exact chain line, but only an approximation, because we assumed straight lines for our model. Difficult images, where the chain lines are densely covered with ink, the paper is in an abraded condition, or when lines in the border area of the image are only partly depicted, can lead to false positives or false negatives. Under very difficult image conditions, the application of DSAC can lead to inaccurate line predictions, e.g., if a too large bounding box size is determined by our method or the estimated rotation angle is not accurate enough. In these cases, the bounding box might contain line segments or noise that do not belong to the actual line. Difficult cases need to be reviewed by art technologists, but our method achieves a high success rate such that it can greatly support the art technologists in their analysis of the artworks.

## 5. Conclusions

We presented an end-to-end trainable deep learning method for chain line segmentation and parameterization in historical prints. In the experiments, we showed that our ChainLineNet achieves the best visual and quantitative chain line detection results for our historical print dataset. Moreover, the comparison of the automatically computed chain line distance intervals with the manually measured distance intervals by an art technologist shows a low error of less than 0.7 mm. The high accuracy and reliability of our method give the opportunity to automatically compare the chain line distances of a larger number of historical prints in order to draw conclusions about the origin of the papers. Thus, our automatic deep-learning-based method can be very beneficial to support the art historical and technological research of museums and libraries. Future work could build on the automatic chain line detection and distance computation to extract chain line distance patterns and perform a similarity search to identify moldmates.

## Figures and Tables

**Table 1:** Evaluation of pixelwise precision, recall, and the Dice coefficient for chain line segmentation of the validation set with 12 images. Best scores are highlighted in bold.

Method	Precision	Recall	Dice Coefficient
UNet (F =16)	0.4046	0.5070	0.4464
UNet (F =64)	0.3958	0.5034	0.4392
UNet-GAN (F =16)	**0.4283**	0.4787	0.4437
UNet-GAN (F =64)	0.3829	0.4591	0.4108
ResNet-E-D (F =64)	0.3855	0.5935	0.4628
ResNet-GAN (F =64)	0.3920	**0.6001**	**0.4696**

**Table 2:** Evaluation of precision, recall, and the $F_{1}$ score of chain line detection for the test set with 48 images. The number of true positives (TP), false positives (FP), and false negatives (FN) are determined based on a distance threshold of 50 pixels between the predicted and ground truth lines. Best scores are highlighted in bold.

	Number of Lines	TP	FP	FN	Precision (%)	Recall (%)	$F_{1}$ Score (%)
Ground truth (manually annotated)	342	342	0	0	100.00	100.00	100.00
Reference (manually measured)	339	339	0	3	**100.00**	**99.12**	**99.56**
PatchDeepHough	528	307	221	35	58.14	89.77	70.57
PatchUNet-Hough	228	175	53	162	76.75	51.93	61.95
PatchUNet-RANSAC	325	305	20	32	93.85	90.50	92.15
ChainLineNet-1 (BCE+DICE+DSAC)	323	315	8	27	97.52	92.11	94.74
ChainLineNet-2 (BCE+DICE)	330	322	8	20	97.58	94.15	95.83
ChainLineNet (BCE+DICE+DSAC+MLE)	333	327	6	15	**98.20**	**95.61**	**96.89**

## Data Availability

The data used for the development and evaluation of the method was collected within the research project “Critical catalogue of Luther portraits (1519–1530)” by the Germanisches Nationalmuseum Nürnberg, FAU Erlangen-Nürnberg and TH Köln, in which A.S., T.K., A.M., and V.C. are involved. The photographs of the historical prints were captured by T.K., all rights reserved by the respective museum/library. The image data of the research project can soon be viewed online at the Cranach Digital Archive (https://lucascranach.org/).
